# Endoscopic 1½-transseptal approach for pituitary surgery

**DOI:** 10.3389/fonc.2022.1116408

**Published:** 2023-01-12

**Authors:** Zixiang Cong, Junhao Zhu, Huaiyu Sun, Chao Tang, Jin Yang, Chiyuan Ma

**Affiliations:** ^1^ Department of Neurosurgery, Affiliated Jinling Hospital, Medicine School of Nanjing University, Nanjing, Jiangsu, China; ^2^ Department of Neurosurgery, Tiemei General Hospital of Liaoning Province Healthy Industrial Group, Tieling, Liaoning, China

**Keywords:** endoscopic transseptal approach, one-and-a-half nostril endoscopic transsphenoidal approach, pituitary surgery, sinonasal quality of life, skull base

## Abstract

**Background:**

We previously introduced the one-and-a-half (1½) nostril endoscopic transsphenoidal approach (OETA) to reduce the damage to the nasal structures. Here, we reported the modified approach which is called the endoscopic 1½-transseptal approach (EOTA) for pituitary surgery by combining the OETA and the microscopic transseptal approach to simplify intranasal procedures and protect nasal mucosa. In EOTA, we removed the sellar lesions in a corridor that is composed of the right submucosal space and the anterior left ½ nasal cavity.

**Methods:**

We introduced EOTA with a detailed technical description and preliminary clinical outcomes. A total of 128 patients who underwent EOTA for pituitary surgery from July 2018 to September 2020 were reviewed for evaluation of the safety and efficacy of this approach.

**Results:**

EOTA had a high gross total resection (GTR) rate and a 1ow complication rate. GTR was achieved in 106 (82.8%) patients, with 81.4% for pituitary adenomas and 93.3% for other non-adenomatous lesions. Post-operative complications included 3 patients (2.3%) with postoperative cerebrospinal fluid leak, 3 patients (2.3%) with diabetes insipidus, 5 patients (3.9%) with anterior pituitary insufficiency and 2 patients (1.6%) with meningitis. In addition, EOTA simplified the intranasal procedures, which led to shortened operation time (67.8 minutes). The results of ASK nasal-12, the Lund-Kennedy score, and the odor identification test showed that patients who underwent EOTA recovered quickly after surgery and the nasal cavity returned to the preoperative condition both apparently and physiologically one month after surgery.

**Conclusions:**

EOTA is a simple, safe and effective approach for pituitary lesions, which provides not only a sufficient surgical corridor for 2-surgeon/4- or 3-hands technique but also minimally invasive access to the sellar region.

## Introduction

The transsphenoidal approach for pituitary surgery evolved persistently in the past hundred years to a safe and efficient approach to remove lesions.

With the panoramic view in endoscopy, there has been a shift from the traditional microscopic transsphenoidal approach to the endoscopic approach (1). Although the endoscopic endonasal approach is a minimally invasive operation (2), a considerable number of patients suffer from sinonasal comorbidities, such as hyposmia, nasal crusting and discharge, which are associated with greatly reduced quality of life (3).

In order to improve sinonasal quality of life, the endoscopic transsphenoidal approach keeps evolving by borrowing the advantages of other surgical approaches, such as the microscopic transseptal surgery, which reaches the sellar area through the mononostril submucosal transseptal area (4).

Inspired by this approach, several modified endoscopic transseptal approaches have been reported (5–8). However, these modified approaches still require bilateral sub-mucoperichondrial and sub-mucoperiosteal dissection.

In 2016, our team proposed the one-and-a-half (1½) nostril endoscopic transsphenoidal approach (OETA) which maintains sufficient surgical freedom and retains as much nasal septal mucosa as possible (9–11).

Here, we reported a combination approach of OETA and the transseptal approach, which is called the endoscopic 1½-transseptal approach (EOTA), and presented the clinical results of 128 patients who underwent EOTA for pituitary surgery to evaluate the efficiency and the safety of this approach.

## Material and methods

### Patients and study design

We retrospectively reviewed the patients who underwent EOTA for pituitary surgery at Jinling hospital from July 2018 to September 2020. This study was approved by the Institutional Ethics Committee of Jinling Hospital.

The medical records were reviewed for patient demographics, tumor characteristics, operative data, surgical outcomes (surgical complications, hormone results, and visual outcomes), and sinonasal comorbidity. The patients were typically followed at 2 weeks, 1 month, 3 months, 6 months, 12 months after surgery, and annually thereafter for MRI scanning, endocrinological examinations, and other necessary tests.

The Knosp grade (12), tumor size (microadenoma (<10 mm), macroadenoma (between 10 and 40 mm), and giant adenoma (>40 mm)), and gross total resection (GTR, according to the MRI at post-op 3 months) were evaluated by the pre-/post-operative MRI scanning (T2-weighted and T1-weighted sequences with and without gadolinium enhancement).

The endocrinological type of adenomas, which is now referred to as pituitary neuroendocrine tumor (PitNET) (13), the anterior pituitary insufficiency, and biochemical remission were assessed by the pre-/post-operative endocrinological examinations. The determination of biochemical remission varies depending on the tumor type. For prolactinomas, the serum prolactin level should be within the normal range. For growth hormone-secreting adenomas, the criteria are suppression of growth hormone secretion of less than 1.0 ng/ml during an oral glucose tolerance test and a normal value of IGF-1. Remission of the adrenocorticotropic hormone-secreting adenomas was defined as the disappearance of hypercortisolism, with normal basal plasma cortisol level after surgery.

All patients underwent pre-operative and post-operative ophthalmological examinations (visual acuity and visual field) at 3 months of follow-up to assess the visual outcomes.

Sinonasal morbidity was evaluated by the questionnaire Anterior Skull Base Nasal Inventory-12 (ASK Nasal-12) (14) and Lund Kennedy score (15) under endoscopic assessment at baseline, 2 weeks, 1 month, and 3 months after surgery. The objective olfactory function was assessed by the odor identification test using Sniffin’ Sticks at baseline, 2 weeks, 1 month, and 3 months after surgery, which is composed of sixteen multiple forced choices from a list of four descriptors based on pen-like odor-dispensing devices (16). It has been used and validated in endoscopic skull base surgery to assess patients’ olfaction (17, 18).

### Surgical technique

All the surgical procedures were performed by 2 neurosurgeons (ZX Cong and CY Ma). The patient is in the supine position with the head in a neutral position. The bilateral nasal cavities were packed with cottonoids containing decongestants (1:10,000 epinephrine) for several minutes. The right nostril was routinely used as the main surgical corridor with a 0° or 30° endoscope (Karl Storz, Tuttlingen, Germany). The anatomy of the nasal septum was examined in case of serious deviation or deformity. It is not necessary to compress and out-fracture the inferior and middle turbinates laterally for enlarging the nasal corridor because the instruments reach the sellar region through the submucosal space.

**Figure 1:**
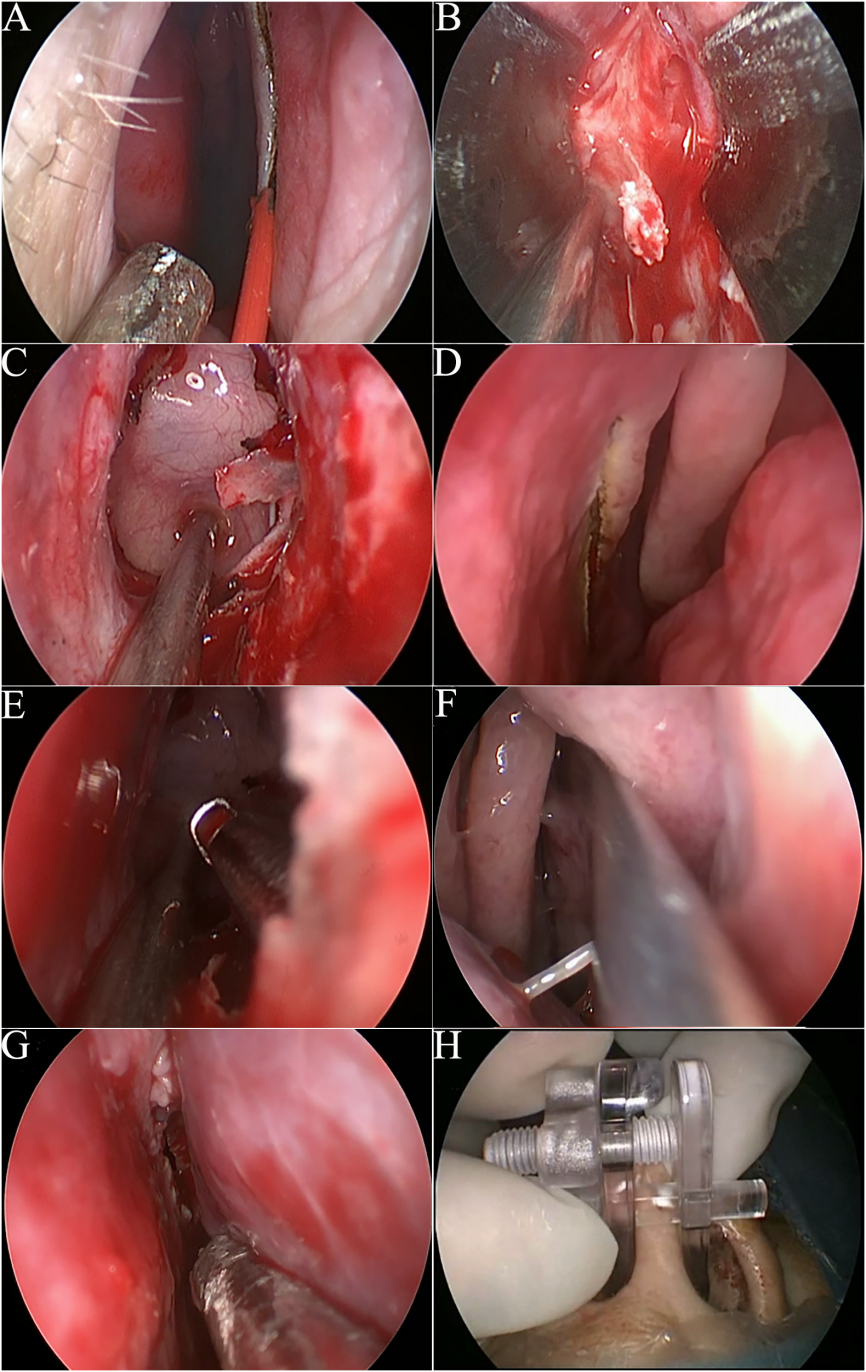
Intraoperative images of surgical procedures. **(A)** The incision of the right nasal cavity. **(B)** The nasal speculum was inserted into the level of sphenoidal rostrum. **(C)** The shaped transseptal corridor. **(D)** The incision of the left nasal cavity. **(E)** 2-surgeon/4 or 3-hands manipulation from bilateral incisions. **(F-H)**. The right and left nasoseptal mucosal flaps were replaced and fixed by a nasal septal retainer.

The Killian incision was made in the right nasal cavity with unipolar electrocautery (**Figure 1A**) and sub-mucoperichondrial dissection of the cartilaginous septum was performed under endoscopic control. Then, sub-mucoperiosteal dissection was continued in the sub-periosteal plane as far as the sphenoid rostrum on the right side of the bony septum. The partial posterior bony septum (the vomer and the inferior part of the perpendicular plate of the ethmoid) was removed to expose the left sphenoidal rostrum.

The nasal speculum was inserted from the right septal incision to create a robust operating space reaching the sphenoid sinus (**Figure 1B**). The anterior wall of the sphenoid sinus was opened by a high-speed drill through the working space between the blades of the speculum. The intersphenoid sinus septum was also resected. After sphenoidotomy, the speculum was removed from the right nostril. The use of the speculum lasted only for a few minutes, but it enabled the long time maintenance of the transseptal corridor (**Figure 1C**).

**Figure 2:**
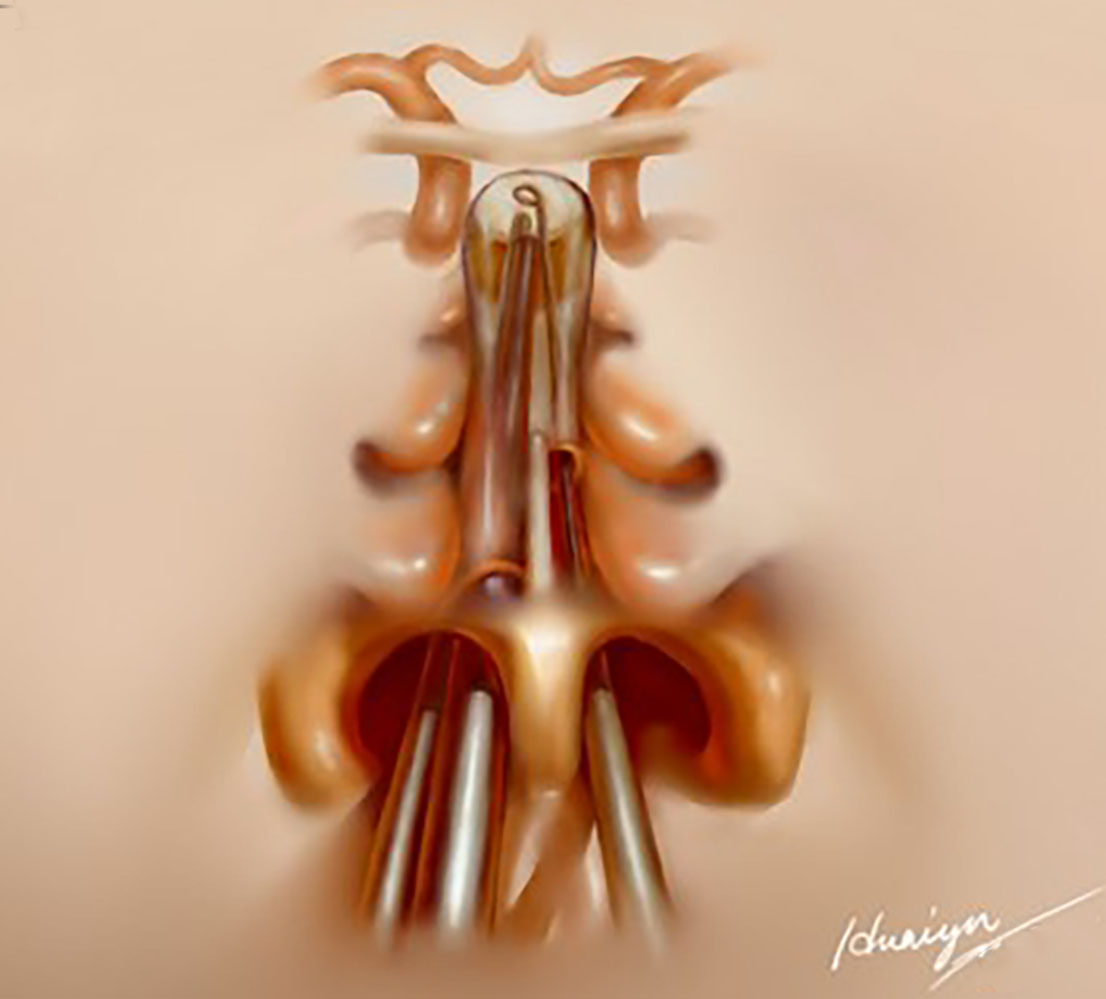
Schematic illustration of endoscopic 1½-transseptal approach.

The following procedure was similar to that of the OETA as we described before (9). Briefly speaking, a small vertical incision of the left septal mucosa was made at the anterior level of the left middle turbinate (**Figure 1D**). Then, the instruments could be inserted into the right transseptal corridor from the incision in the left nostril and access the operating field, allowing access for two surgeons using the 3/4- handed technique (**Figure 1E**). The assistant surgeon guided the endoscope through the right nostril while the senior surgeon manipulated the instruments through both nostrils. A schematic illustration of EOTA was shown in **Figure 2**.

After tumor resection and sellar reconstruction, the septal mucosa was replaced and fixed by a nasal septal retainer (**Figures 1F–H**). No nasal packing was performed. On the second day after surgery, the retainer was removed.

### The sellar reconstruction in EOTA

Under most circumstances, the artificial dura mater and fibrin sealant were used to repair the defects in the skull base. The right dissected septal mucosa could be converted to the Hadad-Bassagasteguy flap (HBF) (19) when a high-flow intraoperative cerebrospinal fluid (CSF) leakage occurred. When the HBF was needed, the superior septal incision was made from 2 to 3 mm below the sphenoid ostium to the superior end of the right incision. Then, the inferior septal mucosa was cut along the floor of the nasal cavity from the choanae to the inferior end of the right vertical incision of EOTA.

## Results

A total of 128 patients with complete medical records and follow-up results were included in this study. The mean time of follow-up was 14 months (ranging from 10 months to 24 months). The characteristics of patients were summarized in **Table 1**. Pathologic diagnoses included 113 (88.3%) pituitary adenomas, 8 Rathke cleft cysts (6.3%), 2 craniopharyngioma (1.6%), 2 chordoma (1.6%), 2 pituitary abscess (1.6%), and 1 pituicytoma (0.8%).

**Table 1:** Patient characteristics and the tumor resection results.

Demographics and characteristics	N (%)	GTR, n (%)
Age (yrs)	53	
Sex, male/female	59/76	
Tumor type		
Pituitary adenoma	113 (88.3%)	92 (81.4%)
Endocrinological type		
FPA	37 (28.8%)	32 (86.5%)/29 (75.7%)*
NFPA	76 (59.4%)	60 (78.9%)
Knosp grade		
Knosp grade ≤ 2	75 (58.6%)	69 (92%)
Knosp grade ≥ 3	38 (29.7%)	23 (60.5%)
Tumor size		
Micro-adenoma	10 (7.8%)	10 (100%)
Macro-adenoma	94 (73.4%)	88 (93.6%)
Giant-adenoma	9 (7.0%)	5 (55.6%)
Others	15 (11.7%)	14 (93.3%)
Rathke’s cleft cyst	8 (6.3%)	8 (100%)
Craniopharyngioma	2 (1.6%)	2 (100%)
Chordoma	2 (1.6%)	1 (50%)
Pituitary abscess	2 (1.6%)	2 (100%)
Pituicytoma	1 (0.8%)	1 (100%)
**Total**	128	106 (82.8%)

* Remission rate.

GTR, gross total resection.

Of the 113 pituitary adenoma patients, 37 cases were functioning pituitary adenomas (FPA) and 76 cases were non-functioning pituitary adenomas (NFPA). According to the preoperative MRI scanning, 10 (7.8%) cases were classified as micro-adenomas, 94 (73.4%) cases as macro-adenomas, and 9 (7.0%) cases as giant adenomas. Based on the Knosp classification, there were 75 (58.6%) cases with Knosp grade ≤ 2 and 38 (29.7%) cases with Knosp grade ≥ 3.

### The safety and efficacy of EOTA

The mean operative time in these series was 67.8 minutes and the mean length of stay in the hospital was 6.9 days.

The gross total resection (GTR) was achieved in 106 (82.8%) patients (detailed results were shown in **Table 1**). The GTR rate was 81.4% (92/113) for patients with pituitary adenomas and 93.3% (14/15) for patients with other non-adenomatous lesions. The GTR rate was 92% in adenomas without cavernous sinus invasion (Knosp grade ≤ 2) and 60.5% in adenomas with cavernous sinus invasion (Knosp grade ≥ 3). GTR was achieved in all the micro-adenomas, in 93.6% of macro-adenomas, and 55.6% of giant adenomas.

Hormonal remission, determined by repeated post-operative serum hormone detection, was achieved in 75.7% (29/37) cases of FPA at the last follow-up. (**Table 2**)

**Table 2:** Post-operative surgical outcomes.

Post-operative surgical outcomes	n (%)
**Endocrine outcomes**	
Remission Failure	29 (75.7%) 8 (24.3)
**Surgical complications**	
Intraoperative CSF leak	49 (38.3%)
Postoperative CSF leak Meningitis	3 (2.3%) 2 (1.6%)
Diabetes insipidus	3 (2.3%)
Anterior pituitary insufficiency	5 (3.9%)
ICA Injury	0 (0%)
Death	0 (0%)
**Visual outcomes**	
Improved Unchanged Worthened	55 (82.1%) 12 (17.9%) 0 (0%)

**Figure 3:**
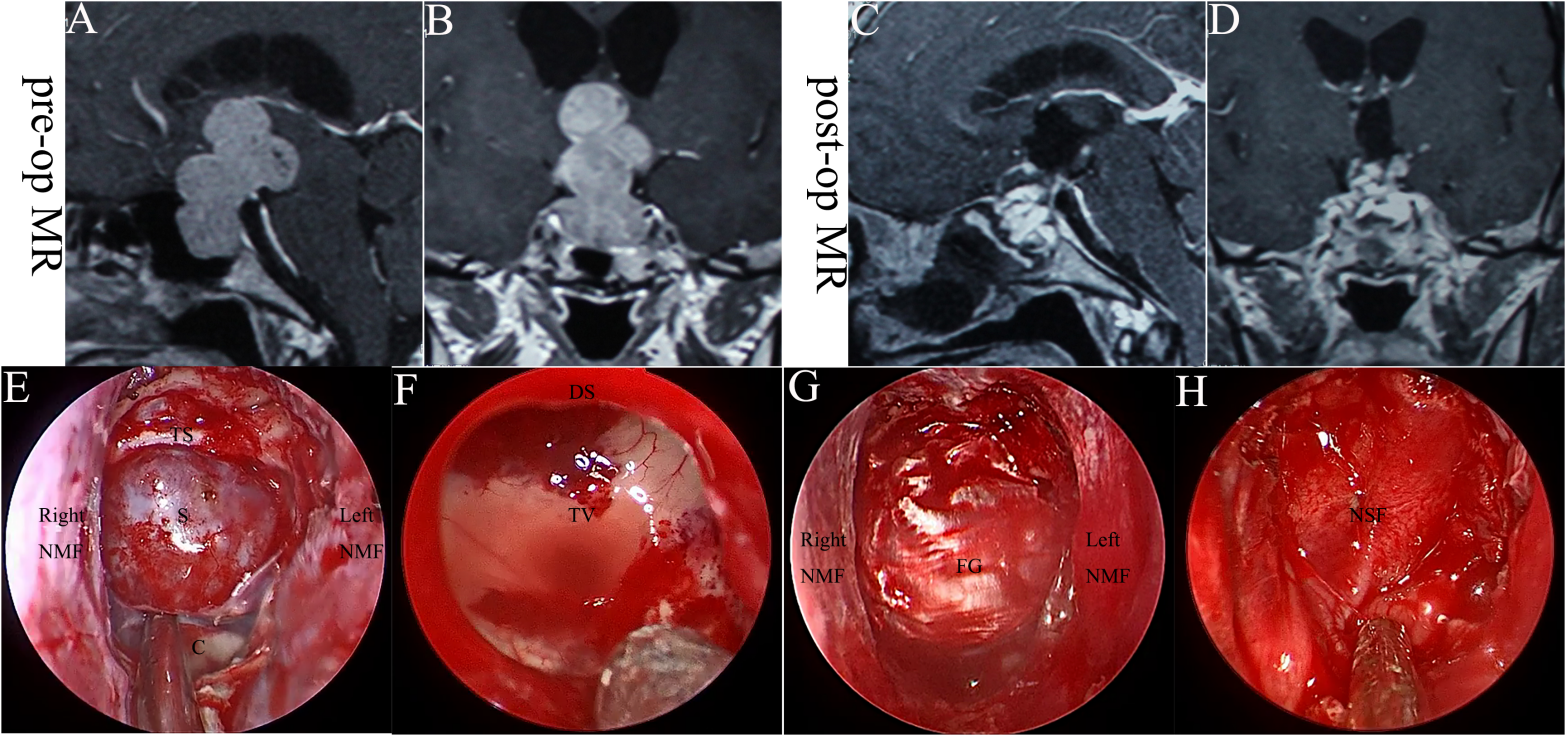
The case with high-flow CSF leak was reconstructed with a nasoseptal flap. **(A, B)**. Pre-operative MRI scanning. **(C, D)**. Post-operative MRI scanning. **(E–H)**. Intraoperative images. S, sella; C, clivus; TS, tuberculum sellae; NMF, nasoseptal mucosal flap; DS, diaphragma sellae; TV, the third ventricle; FG, fascial graft; NSF, nasoseptal flap.

In regards to the surgical complications (**Table 2**), the intraoperative CSF leakage was encountered in 49 (38.3%) patients, and 4 patients with high-flow CSF leak (Kelly grade 3) were reconstructed with a nasoseptal flap. A representative case was shown in **Figure 3**. The nasoseptal flap was used in the reconstruction phase when the intraoperative high-flow CSF leakage was confirmed. There were 3 (2.3%) patients with postoperative CSF leakage (two giant adenomas with Knosp grade 4 and one chordoma) and all of them recovered after lumbar drainage placement. Two of the three patients developed meningitis and all recovered after antibiotic therapy. Other postoperative complications included diabetes insipidus in 3 (2.3%) cases (all recovered after 6 months) and anterior pituitary insufficiency (taking hormone replacement therapy) in 5 (3.9%) cases. No ICA Injury or death happened in this series.

More than half of the patients (52.3%, 67/128) suffered from decreased visual acuity and visual field defects before surgery. Most of the patients (82.1%, 55/67) had improved visual status and no patient presented a worsened visual status. (**Table 2**)

### Sinonasal quality of life

The main sinonasal complaints at 2 weeks after surgery were: headache (15.6%), nasal discharge (11.7%), trouble breathing (10.2%), hyposmia (7.8%), nasal synechia (2.3%) and epistaxis (0.8%). One month after surgery, most symptoms disappeared and were relieved with 4.7% of patients complaining about nasal discharge, followed by hyposmia (3.9%), trouble breathing (3.1%), and headache (2.3%). At 3 months of follow-up, nearly all these nasal symptoms disappeared. (**Table 3**)

**Table 3:** Sinonasal comorbidity after surgery.

Sinonasal comorbidity		preoperatively	2 weeks after surgery	1 month after surgery	3 month after surgery
Symptoms	headache	2 (1.6%)	20 (15.6%)	3 (2.3%)	1 (0.8%)
	hyposmia	1 (0.8%)	10 (7.8%)	5 (3.9%)	1 (0.8%)
	nasal discharge	1 (0.8%)	15 (11.7%)	6 (4.7%)	0 (0%)
	trouble breathing	0 (0%)	13 (10.2%)	4 (3.1%)	0 (0%)
	epistaxis	0 (0%)	1 (0.8%)	0 (0%)	0 (0%)
	septal hematoma	0 (0%)	0 (0%)	0 (0%)	0 (0%)
	septal perforation	0 (0%)	0 (0%)	0 (0%)	0 (0%)
	synechia	0 (0%)	3 (2.3%)	0 (0%)	0 (0%)
ASK nasal-12		2.43 ± 2.79	21.56 ± 4.32	7.45 ± 2.45	2.78 ± 1.97
Lund Kennedy score		0.87 ± 0.14	3.18 ± 1.21	1.19 ± 0.45	0.91 ± 0.33
Odor identification test using Sniffin’ Sticks		12.89 ± 2.46	8.23 ± 1.10	10.13 ± 3.15	12.67 ± 2.29

As for the sinonasal quality of life assessed by the ASK nasal-12, the scores increased two weeks after surgery and significantly decreased 1 month after surgery. Three months after surgery, the scores returned to the baseline. The Lund-Kennedy score showed that after one month, the nasal cavity of patients who underwent EOTA had recovered to the baseline. The odor identification test also showed that the patients had the same olfactory performance as the baseline one month after surgery. (**Table 3**)

## Discussion

In this study, we described the surgical technique and clinical application of EOTA, which was a combination of the OETA and the transseptal approach. Our team reported the OETA in 2016, which completely preserved the mucosa in the left nostril, especially the septal olfactory strip and the vascular pedicles of sphenopalatine, and reduced the risk of anosmia and epistaxis (9). The randomized controlled trial comparing the OETA and binostril approach showed that the OETA group had better olfaction function than the binostril group one month after surgery (11).

During the application of OETA, we modified this approach by learning from the transseptal approach and designed EOTA, which takes advantage of OETA and the transseptal approach.

Compared with OETA, the prominent advantages of EOTA are that EOTA has a simplified intranasal procedure and protects the mucosa of the right nostril.

Without the procedures of enlarging the nasal corridor, the mean operation time in this series (67.8 minutes) is much shorter than that of OETA (usually more than 1.5 hours), which requires more complex intranasal procedures to establish a nasal corridor. Of note, the operating time of EOTA was also shorter than other published endoscopic transseptal approaches. (**Table 4**) This could also be explained by the improved skills and expertise of the surgeons.

**Table 4:** Overview of surgical outcomes of published pituitary adenoma surgery.

Reference	Cases	Approach	OT (min)	GTR	Csf	Di	Pi	Me
Paluzzi et al. (20)	555	transnasal	/	65.3%	5%	2.5%	3.1%	0.9%
Wang et al. (21)	1166	transnasal	/	91.7%	0.6%	0.7%	1.29%	1%
Magro et al. (22)	300	transnasal		59%	2.7%	6.2%	13.7%	3.3%
Wen et al. (9), (OETA)	57	transnasal	/	79%	3.5%	5.3%	5.3%	/
Kim et al. (23)	331	transnasal	/	74.9%	2.4%	3%	/	5.4%
Anik et al. (24)	401	transnasal	/	77.56%	2.7%	3%	7.9%	0.2%
Li et al. (25)	2032	transnasal	/	80.1%	1.7%	2.08%	3.5%	1.5%
Tewfik et al. (8)	23	transseptal	152.6	/	4.3%	/	/	/
Hong et al. (5)	51	transseptal	144.6	72.5%	0%	/	/	/
Favier et al. (26)	119	transseptal	98	48.8%	3%	0.8%	3%	1.7%
Current series	113	transseptal	67.8	81.4%	2.3%	2.3%	3.9%	1.6%

OT, Operation time; GTR, Gross total resection; Csf, Cerebrospinal fluid leak; Di, Diabetes insipidus; Pi, Pituitary insufficiency; Ep, Epistaxis; Me, Meningitis.

Besides a shorter operating time, EOTA maximally protected the natural nasal structures. During operation, it is not necessary to out-fracture or resect the inferior and middle turbinates laterally for enlarging the nasal corridor even in patients with narrow nasal spaces caused by anatomic variations (e.g. deviation of nasal septum and septal spur), because surgeons operate in the transseptal corridor instead of transnasally. What’s more, the incisions of EOTA are far away from the sphenopalatine or posterior nasal artery, as well as the septal olfactory strip. Therefore, EOTA has fewer early-time nasal symptoms than the endoscopic transnasal approach, which was similar to other reported endoscopic transseptal approaches (5, 27).

In this study, we provided preliminary evidence that EOTA was a safe and effective approach for pituitary surgery. EOTA provides a wide surgical corridor and allows binostril access with the 2-surgeon/3- or 4-hands technique.

How to maintain a wild and robust working space is the key technical point of the endoscopic transseptal approach. A stable working space between bilateral nasal septal mucosa is a prerequisite for ensuring the maneuverability of the instruments. Many methods have been reported, such as a tagging suture between the septal mucosa and vestibule (5) or using a nasal speculum (7). However, suturing may tear the vulnerable mucosa and the hard blades of the nasal speculum severely restrict the lateral movement of the endoscope and instruments. In clinical practice, we found that short-time application (3-5 min) of the speculum could shape and maintain the surgical corridor for a long time. This is a simple and effective technique. If the speculum is not available, packing with cottonoids containing decongestants is also effective in shaping and maintaining the surgical corridor.

The operative maneuverability of EOTA can also be reflected by the GTR and complication rates. We summarized the GTR and complication rates of the endoscopic transnasal/transseptal approaches for pituitary lesions in several published series (**Table 4**). The GTR rate of EOTA, which is at a high level among the endoscopic transnasal approaches (20–25), is higher than other endoscopic transseptal approaches (5, 8, 26). The GTR rates in our and Hong’s series (binostril working) (5) were higher than that of Favier’s series (26) (mononostril working), which suggested surgeons could better apply the 2-surgeon/4- or 3-hands technique by the binostril transseptal approach compared with the mononostril transseptal approach. The complication incidence in our series was similar to the published data (5, 20–26).

The sinonasal quality of life has become a more and more important topic in endoscopic skull base surgery. EOTA was introduced with the aim of reducing sinonasal morbidity and improving sinonasal quality of life. The results of ASK nasal-12, the Lund-Kennedy score, and the odor identification test showed that patients who underwent EOTA recovered quickly after surgery and the nasal cavity returned to the preoperative condition both apparently and physiologically.

In addition, the nasal septal retainer is very useful for fixing dissected nasoseptal flaps in most transseptal approaches. Application of the retainer produced a significant reduction in pain and nasal obstruction compared with nasal packing (28).

### Limitations of EOTA

For sellar tumors with markedly lateral extension, which often need expanded approaches, the working space in EOTA was limited to expose the lateral extension of the surgical field, in addition to the inability to add transpterygoid exposure.

## Conclusion

EOTA is a simple, safe and effective approach for pituitary surgery, which provides not only a sufficient surgical corridor for 2-surgeon/4- or 3-hands technique but also minimally invasive access to the sellar region.

## Data availability statement

The raw data supporting the conclusions of this article will be made available by the authors, without undue reservation.

## Ethics statement

The studies involving human participants were reviewed and approved by Institutional Ethics Committee of Jinling Hospital. The patients/participants provided their written informed consent to participate in this study.

## Author contributions

CM designed the approach. CM and ZC performed the surgery. JZ and ZC are responsible for data collection and quality control. JZ and ZC drafted the manuscript. JY and CT are responsible for revising and finalizing this paper. All authors contributed to the article and approved the submitted version.
